# Modified technique for thermal radiofrequency ablation of Thoracic dorsal root ganglia under combined fluoroscopy and CT guidance: a randomized clinical trial

**DOI:** 10.1186/s12871-019-0906-4

**Published:** 2019-12-18

**Authors:** Raafat M. Reyad, Hossam Z. Ghobrial, Ehab H. Shaker, Ehab M. Reyad, Mohammed H. Shaaban, Rania H. Hashem, Wael M. Darwish

**Affiliations:** 1 Department of Anesthesia & Pain Management, National Cancer Institute, Cairo University, Cairo, Egypt; 2 Department of Clinical Pathology, National Hepatology and Tropical Medicine Research Institute, Cairo, Egypt; 3 Department of Diagnostic & Interventional Radiology, Faculty of Medicine, Cairo University, Cairo, Egypt; 4 Department of Diagnostic & Interventional Radiology, National Cancer Institute, Cairo University, Cairo, Egypt

**Keywords:** Thermal radiofrequency ablation, Intractable pain, Chest malignancies, Transforaminal approach, Dorsal root ganglia, Suprapedicular approach

## Abstract

**Background:**

This study is comparing thermal radiofrequency ablation (TRFA) of the thoracic dorsal root ganglia (TDRG) guided by Xper CT and fluoroscopy with the standard fluoroscopy.

**Methods:**

This randomized clinical trial included 78 patients suffering from chronic refractory pain due to chest malignancies randomly allocated into one of two groups according to guidance of TRFA of TDRG. In CT guided group (*n* = 40) TRFA was done under integrated Xper CT-scan and fluoroscopy guidance, while it was done under fluoroscopy guidance only in standard group (*n* = 38). The primary outcome was pain intensity measured by visual analog scale (VAS) score, functional improvement and consumption of analgesics. The secondary outcome measures were patient global impression of changes (PGIC) and adverse effects.

**Results:**

VAS scores decreased in the two groups compared to baseline values (*p* < 0.001) and were lower in CT guided group up to 12 weeks. Pregabalin and oxycodone consumption was higher in the standard group at 1, 4 and 12 weeks (*p* < 0.001). Functional improvement showed near significant difference between the two groups (*P* = 0.06 at week 1, 0.07 at week 4 respectively) while the difference was statistically significant at week 12 (*P* = 0.04). PGIC showed near significant difference only at week 1 (*P* = 0.07) while the per-patient adverse events were lower in CT guided group (*p* = 0.027).

**Conclusions:**

Integrated modality guidance with Xper CT-scan and fluoroscopy together with suprapedicular inferior transforaminal approach may improve efficacy and safety of TRFA of TDRG for the treatment of intractable chest pain in cancer patients.

**Trial registration:**

The study was retrospectively registered at clinicaltrials.gov on 04/22/2018 (Registration No.: NCT03533413).

## Background

Thoracic pain represents approximately 3–5% of patient visits to pain clinic worldwide [1]. Lung cancer is one of the three most common malignancies that are highly associated with pain,together with head and neck, breast cancers, and advanced or metastatic diseases increase the prevalence of pain from 51 to 66% in cancer patients [2]. Lung cancer is the most common cancer worldwide; 1.8 million cases are diagnosed annually (13% of all cancers diagnoses) [3]. Post-thoracotomy pain occurs in 30–50% of patients undergoing thoracotomy [4].

Pain is the presenting symptom in 20% of cases of lung cancer and it may be more distressing to patients with lung cancer than to patients with other cancers [5]. Pain affects the patient’s psyche, sleep, behavior and ultimately quality of life. The management of such pain is challenging and it may be either medical or interventional. Interventional treatment of such refractory pain due to lung cancer could be attributed to multiplicity of pain generators (visceral-somatic-neuropathic) such as chest wall pain, costo-pleural syndrome, pancoast tumor, rib metastasis, post-thoracotomy pain syndrome, post-herpetic neuralgia and pain related to diagnostic or therapeutic procedures e.g. chemotherapy or radiotherapy-induced pain. Interventional therapies include epidural or intrathecal drug injection, intercostal nerve block, sympathectomy, rhizotomy, and percutaneous cervical cordotomy (PCC) [6]. Rhizotomy refers to the selective, segmental destruction of the dorsal sensory rootlets to interrupt pain perception by the spinal cord. This could be accomplished using neurosurgical or chemical means or using selective percutaneous procedures, such as cryoanalgesia and radiofrequency (RF) ablation [6].

There are many technical difficulties in approaching the deep-seated thoracic dorsal root ganglia (DRG) through the transforaminal route. The spine is kyphotic, with the tip at T6, and slightly scoliotic to the right side even in normal subjects [7]. Spinous processes are acute, especially at the T5-T8 level. In addition, broad and wide laminae together with narrow intervertebral foramina are also obstacles [8]. The intervertebral foramina are further masked by the facet joints and the crowded nature of the costovertebral and the costotransverse joints [9].

The extra guidance of Xper CT than conventional fluoroscopy may improve the success of the transforaminal approach to thoracic DRG considering all these factors of technical difficulties due to natural anatomical barriers lowering the efficacy of dorsal rhizotomy (which is still the standard interventional therapy for treating lung cancer pain worldwide). The authors hypothesize that combining the Xper CT scan with fluoroscopy to guide RF ablation through the transforaminal route can enhance its efficacy and safety in relieving the intractable pain of chest malignancies. The current study aimed to compare the results of thermal radiofrequency ablation (TRFA) of the thoracic DRG under combined Xper CT - fluoroscopy guidance with the standard fluoroscopy technique.

## Methods

This single-blinded, parallel group, randomized clinical trial was conducted in the National Cancer Institute,Cairo University during the period from April 2017 to March 2018 after obtaining the approval of the Institutional Review Board (approval No.: 201617013. 2P). The study was retrospectively registered at clinicaltrials.gov on 04/22/2018 (Registration No.: NCT03533413). The study fulfilled the principles of the Helsinki Declaration and followed the Medical Research Involving Human Subjects Act (WMO). This study adheres to CONSORT guidelines for reporting clinical trials. The purpose, benefits, possible risks and expectations were explained to all patients before their enrollment in the study and a written informed consent was obtained from each patient. Patients were recruited from the pain clinic. Eligible patients were 18 years or older and suffering from chronic moderate-to-severe pain (VAS score ≥ 40 mm), due to chest malignancies and pain was refractory to the maximally tolerated dose of opioids for at least four weeks [10]. The malignancies included lung cancer, pleural mesothelioma, chest wall tumors and metastatic deposits of the chest. The exclusion criteria were sepsis, coagulopathy, malignant epidural invasion, distorted local anatomy, severe cardiorespiratory compromise, neuropsychiatric illness, history of drug dependence and known allergy to contrast media or the medications used.

### Randomization, allocation, and concealment

Eighty patients were randomly allocated into one of two equal groups. In CT group (*n* = 40) TRFA of the thoracic DRG was conducted under Xper Guided CT fluoroscopy guidance, while the procedure was conducted under fluoroscopy guidance only in the standard group (*n* = 40). The random number list was concealed and checked just before a patient’s allocation by personnel blinded to the study.

### Technique for the fluoroscopy-guided procedure

**Fig. 1:**
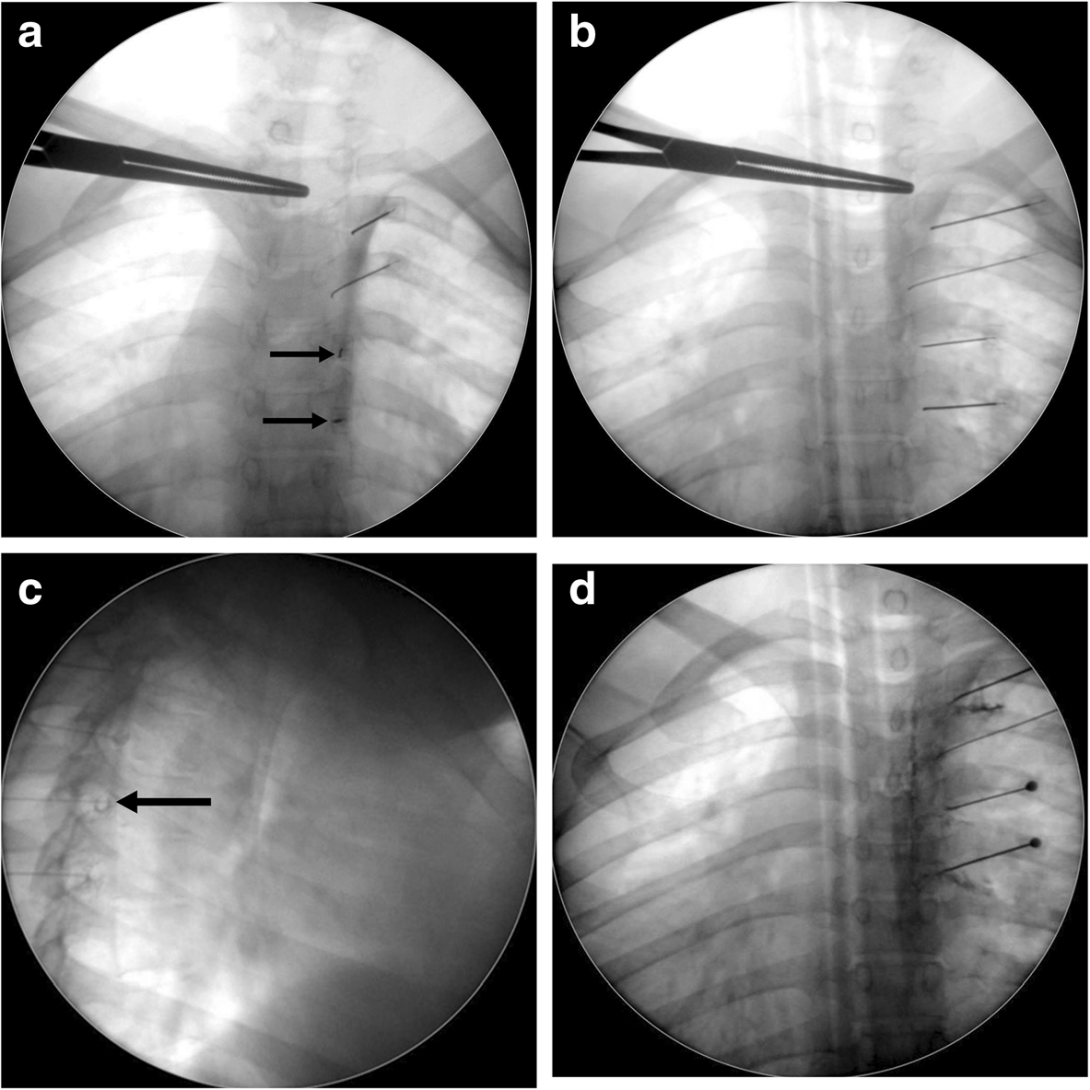
Fluoroscopic pictures showing the technique of thoracic DRG transforaminal approach: **a**) ipsilateral 15° oblique view with 4 RF needles at T3, 4, 5, 6 levels (black arrows point at trajectory-end-on-needles orientation), **b**) P-A view showing the 4 needles tips nearly at the neural foramina, **c**) lateral view showing the RF needles tips in the transforaminal positions after contrast injection (black arrow points at the characteristic signet ring appearance of DRG), **d**) P-A view showing transforaminal, epidural and intercostal spread of the contrast at the targeted levels

The procedure was conducted in the fluoroscopy room where all anesthetic and resuscitation facilities were available. ASA-standard monitors (NIBP, pulse-oximetry, and EKG) were connected to the patient. A G20 intravenous (IV) line was fixed and O_2_ via nasal prongs and a 1 g ceftriaxone (Longacef GSK, Cairo, Egypt) IV infusion was initiated. The procedure was conducted under the ASA recommendation of conscious alert sedation with a 0.5–1.0 μg/kg fentanyl IV (fentanyl citrate 50 μg/mL; Janssen Pharmaceutica, Beerse, Belgium) and a 0.5–1.0 μg/kg dexmedetomidine (Precedex 200 mcg/2 mL, Pfizer, USA) IV in addition to propofol (Diprivan 1%, Fresenius Kabi, USA) IV boluses during TRFA application. The patient was placed prone on a small pillow located under the chest, and the back of the patient was sterilized using 8% povidone iodide and draped. The needle was a Baylis RF needle (100 mm length, 10 mm active tip, curved, G20, sharp needle) (Baylis Medical Company Inc. Montreal, QC Canada). The selected level was checked by history, local examination for rib tenderness and possible neuropathic characters, e.g., allodynia. The fluoroscopic postero/anterior (PA) view was taken and squaring (alignment) of the targeted vertebra was attained by cephalocaudal orientation of the C-arm. An ipsilateral oblique view of 15° was completed and then the port of needle entry was located at the lower 1/3 to 1/4 of the lateral vertebral edge, under the articular pillar and the halo of the transverse process. As a rule, the port of needle entry must be within 4 cm of the midline (Rule of 4) to avoid injuring the parietal pleura [8]. Lidocaine 1% (Debocaine 2%, Sigma-Tec, Egypt) was used for local infiltration of the skin and subcutaneous tissues. The RF cannula was advanced using the trajectory (tunnel) technique with a 15° oblique view and then with a dead-lateral view until the needle tip stopped at the lower- or mid-foraminal zone and behind the central line to avoid segmental blood supply and nerve root injury (Fig. 1). After a negative aspiration for blood, air or CSF, 0.5–1.0 ml of iohexol contrast medium (Omnipaque TM, Nycomed, Ireland) was injected to delineate the dorsal root ganglia, nerve roots, epidural space and the intercostal nerve path (Fig. 1). A thermocouple electrode was inserted and sensory stimulation at 50 Hz and up to 0.5 v and motor stimulation at 2 Hz and up to 1–1.5 v was conducted to verify the needle tip position (tingling paresthesia and/or intercostal muscle contraction inside the needle). Neural mapping of the affected dermatomal (intercostal) levels to be blocked was additionally performed by asking the patient if their original pain was at, above or below the level of sensory/motor stimulation and if paresthesia is concordant with his original pain. Additionally, the impendence was checked (normal range is 150–250 Ω inside the neural foramen). The pain level was checked again after injecting a lidocaine-betamethasone mixture (2 ml of 2% lidocaine/segment and 2 mg/ml betamethasone sodium phosphate plus 5 mg/ml betamethasone dipropionate) (Diprofos 2 mg + 5 mg/ml,MSD/Schering-Plough,NJ,USA). After 2 min, thermal lesioning was conducted using Baylis generator at 80 °C for 120 s twice (up and medial then down and medial to enlarge the lesion size of the DRG), and both sensory and motor stimulation were repeated upon rotation of the needle tip. The patient’s back was dressed and the patient was transferred to the recovery unit where vital signs and pain and neurological findings were checked for 1–2 h before discharge. The patient was instructed clearly to consult the pain team if adverse effects happened, namely, chest pain or dyspnea (pneumothorax) and neurological insults (motor deficits).

### Technique for the combined CT-fluoroscopy guided procedure

**Fig. 2:**
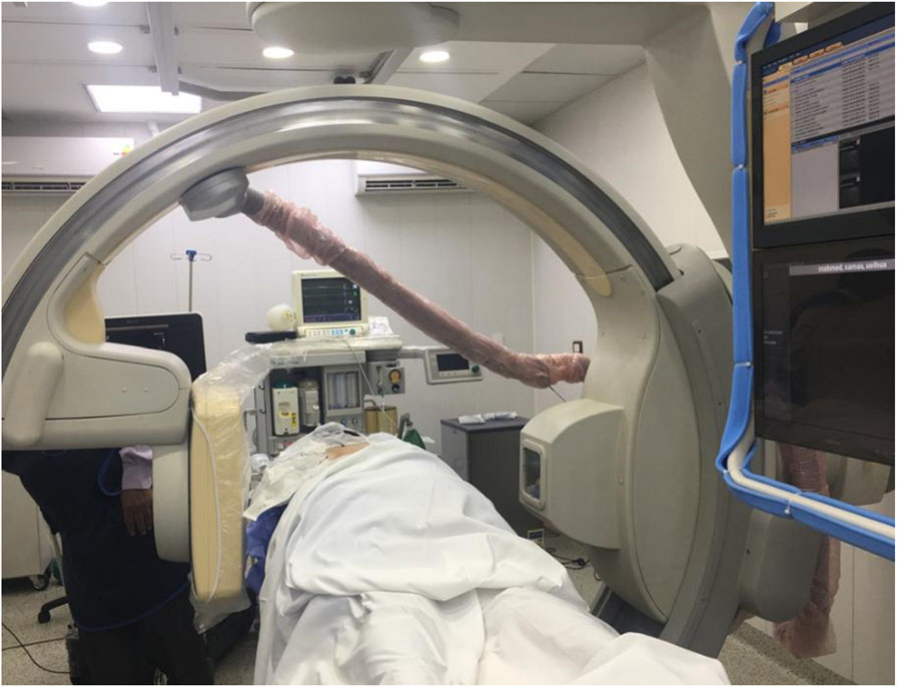
Allura Xper FD 20 Flat detector Fluoroscopy with Xper CT, Philips, Netherlands

**Fig. 3:**
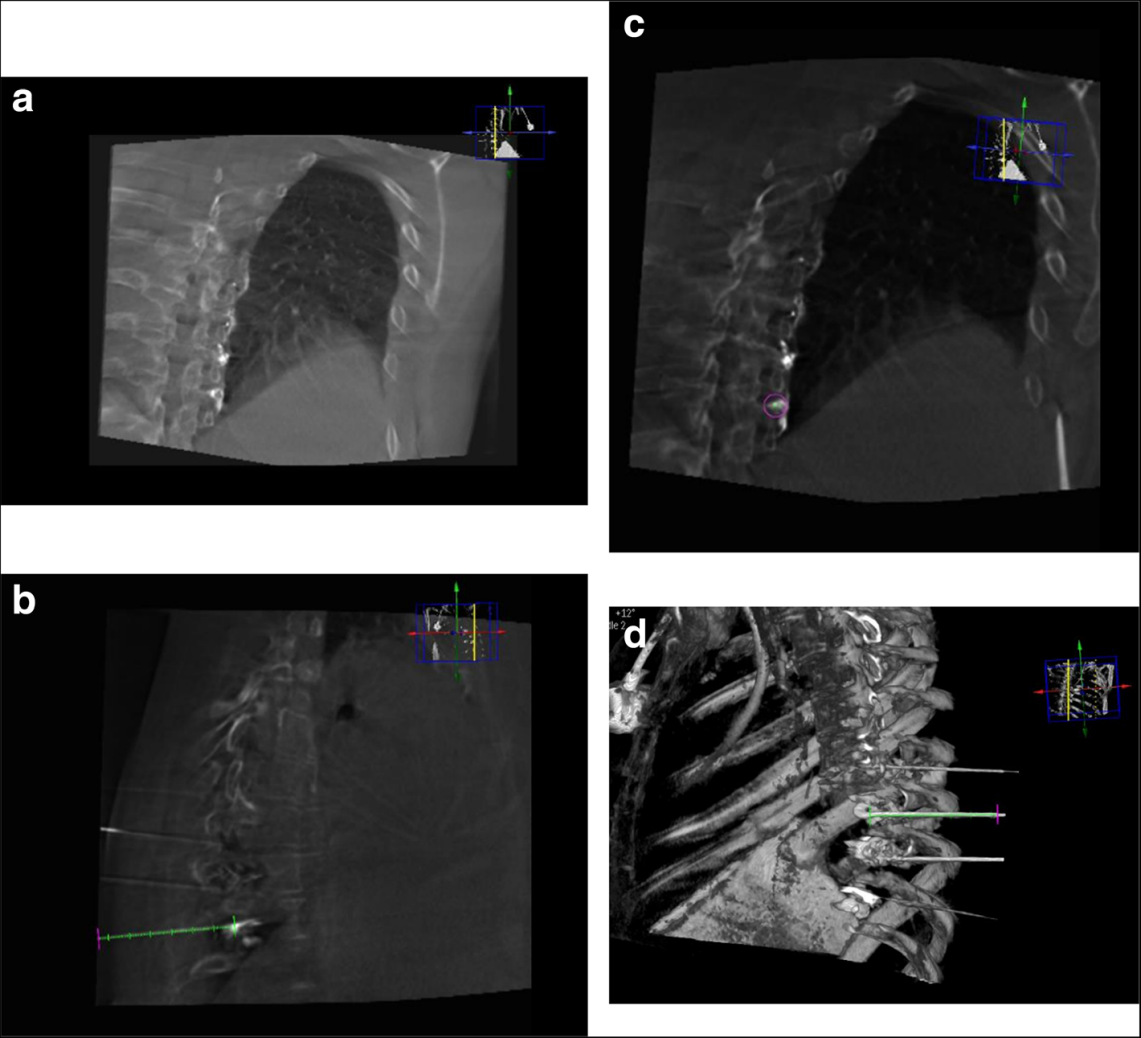
**a, b, c** and **d:** image sequence describing the Xper CT-Fluroscopy technique of thoracic transforaminal approach.

The patient is placed prone on the angio table (Allura Xper FD 20 Flat detector Fluoroscopy with Xper CT, Philips, Netherlands) (Fig. 2) and Xper CT scan of the desired chest levels is performed without contrast to localize the targeted neural foramina with the help of Xper Guide defining the entry point, the needle path and the needle target; thus optimizing the final position of the needle tip. RF stimulation and lesioning are carried out as before, and finally, chest scanogram is performed to rule out pneumothorax (Fig. 3).

In both groups all patients were converted to as needed immediate release oxycodone (Oxynorm® 5- 10 mg Mundipharm) capsules and the average daily required dose to achieve adequate analgesia was calculated then given as sustained release formula (Oxycontin® 10–20-40 mg Mundipharm) in the first visit of the patient after 1 week. Regarding Pregabalin (Lyrica®50–75-150 mg Pfizer), the dose decreased by 25% each week according to patient response with maximum dose 600 mg per day.

A junior clinician of the pain clinic team that was blinded to the study groups was assigned to collect the data. The demographic data included age, gender, chest pathology, body mass index (BMI), duration of the procedure, the side of lesioning, the number of levels/patient, the estimated dose of irradiation exposure (ED), baseline VAS, and medication use. The patient evaluation was conducted 1, 4, and 12 weeks after the procedure. The patients were not allowed to review their previous scores.

The primary outcome measure was pain intensity measured by the visual analog scale (VAS). A 100 mm VAS was presented to the patient as a horizontal line with two ends; the left end represented no pain experienced and the right end represented experiencing the worst pain imaginable. The functional improvement was assessed as a self-reported score after pain procedures representing the percentage of pain reduction; 0–25% means no or minimal improvement, 25–50% means mild improvement, 50–75% means moderate improvement and 75–100% means marked relief [11]. The consumption of analgesics (mg/day) including oxycodone and pregabalin was recorded. The secondary outcome measure was assessed using patient satisfaction with the patient global impression of changes (PGIC) found in Table 1 [12].

**Table 1:** Patient Global Impression of Changes (PGIC)

PGIC	Score
Very much improved	1
Much improved	2
Minimally improved	3
No change	4
Worse	5
Much worse	6
Very much worse	7

The adverse effects were categorized into minor and major groups. The minor events included back pain, soreness, infection, hematoma, and bruises. The major events included pneumothorax, neuritis, motor deficits, and sensory changes, such as discomforting numbness and hypoesthesia, dysesthesia or anesthesia dolorosa.

### Calculation of the sample size

The required sample size was calculated using G*Power Software version 3.1.9 (Universität Düsseldorf, Germany). There were no previous trials comparing the two studied groups of the current trial. The hypothesis was one of “superiority” (that adding the CT to fluoroscopy- guidance would improve the outcome not just matching the traditional approach). We hypothesized that a difference of 3 in the VAS at 12 weeks after treatment would be clinically meaningful. With this difference and a pooled standard deviation of 4, a total of 29 subjects were needed to detect the difference between the two groups at an alpha level of 0.05 and a power of 0.8. Owing to the repeated measures and possible drop outs, the sample was increased by 20% for each condition. Therefore, a sample of 40 patients in each group was recruited in the study.

### Statistical analysis

Data were analyzed using IBM© SPSS© Statistics version 23 (IBM© Corp., Armonk, NY, USA). Data were summarized as a mean and standard deviation or median and range for quantitative data and frequency and percentage for categorical data. The comparisons between quantitative variables were made using the unpaired t-test or the Mann-Whitney test. For comparison of the serial measurements within each group, a mixed linear model was applied. Comparisons of categorical data were made using the Chi-square test or Fisher’s exact test as appropriate. All tests were two-tailed. A *p* value < 0.05 was considered significant.

## Results

**Fig. 4:**
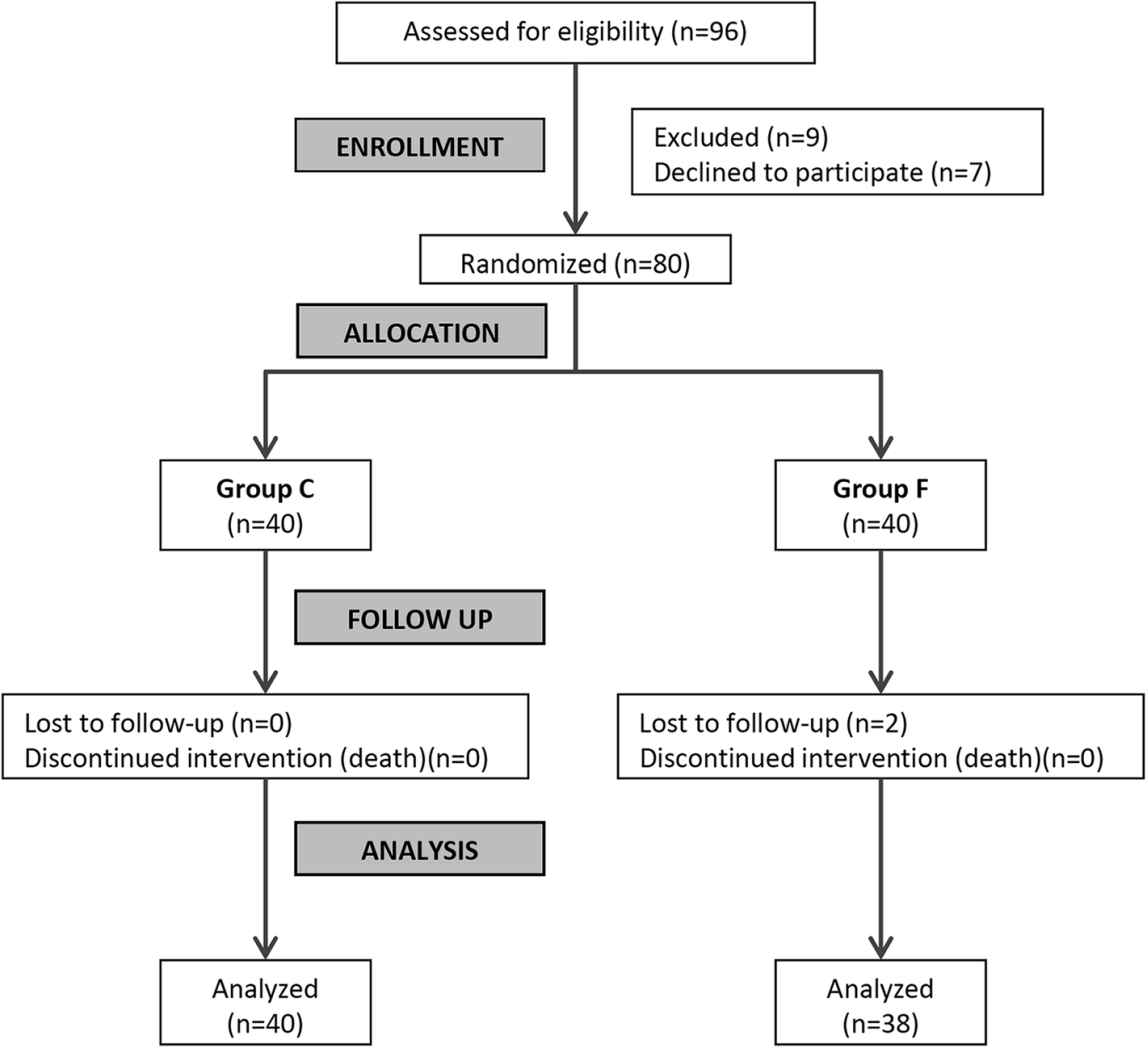
Consort flow chart of the studied groups

Two patients in standard group were lost during the follow-up (Fig. 4). There were no statistically significant differences between the two groups regarding the baseline demographic and clinical characteristics (Table 2) apart from the estimated dose (ED) of irradiation for each injected level, which was highly significant (*p* < 0.001): the CT-guided group was 2 folds higher than the Standard group. The duration of the procedure was significantly longer in the C Group (*p* = 0.037) (Table 3).

**Table 2:** Baseline demographic and clinical characteristics of the two studied groups

	CT-guided group *n* = 40	Standard group *n* = 38	*p* value
Age (years)	57.0 ± 11.7	59.2 ± 13.2	0.438
Gender (male/female)	29/11	25/13	0.521
Chest pathology			
Lung cancer	19 (47.5%)	20 (52.6%)	1.000
Pleural mesothelioma	14 (35.0%)	13 (34.2)	
Chest secondaries	5 (12.5%)	4 (10.5%)	
Chest wall masses	2 (5.0%)	1 (2.6%)	
Body mass index (kg/m2)	29.2 ± 5.2	28.3 ± 4.3	0.409
Side of treatment (Rt/Lt)	21/19	21/17	0.807

Data are supplied as mean ± SD, numbers (%).

**Table 3:** Procedural details in the two studied groups

	CT-guided group *n* = 40	Standard group *n* = 38	*p* value
Duration of procedure (minutes)	26.2 ± 10.7	21.3 ± 9.6	0.037
Number of levels treated/patient			
2 levels	15 (37.5%)	14 (36.8%)	0.862
3 levels	12 (30.0%)	11 (28.9%)	
4 levels	13 (32.5%)	13 (34.3%)	
VAS score before the procedure	72.4 ± 5.2	73.4 ± 4.9	0.385
Oxycodone consumption/day (mg/day)	78 ± 12	80 ± 10	0.428
Pregabalin (mg/day)	311 ± 35	304 ± 23	0.303
Exposure time per level (sec)	21.7 ± 8.1	19.05 ± 6.3	0.112
Estimated dose per level (mGy)	0.28 ± 0.083	0.57 ± 0.23	< 0.001

Data are supplied as mean ± SD, numbers (%).

*VAS* visual analogue scale, mGy = milligray.

**Fig. 5:**
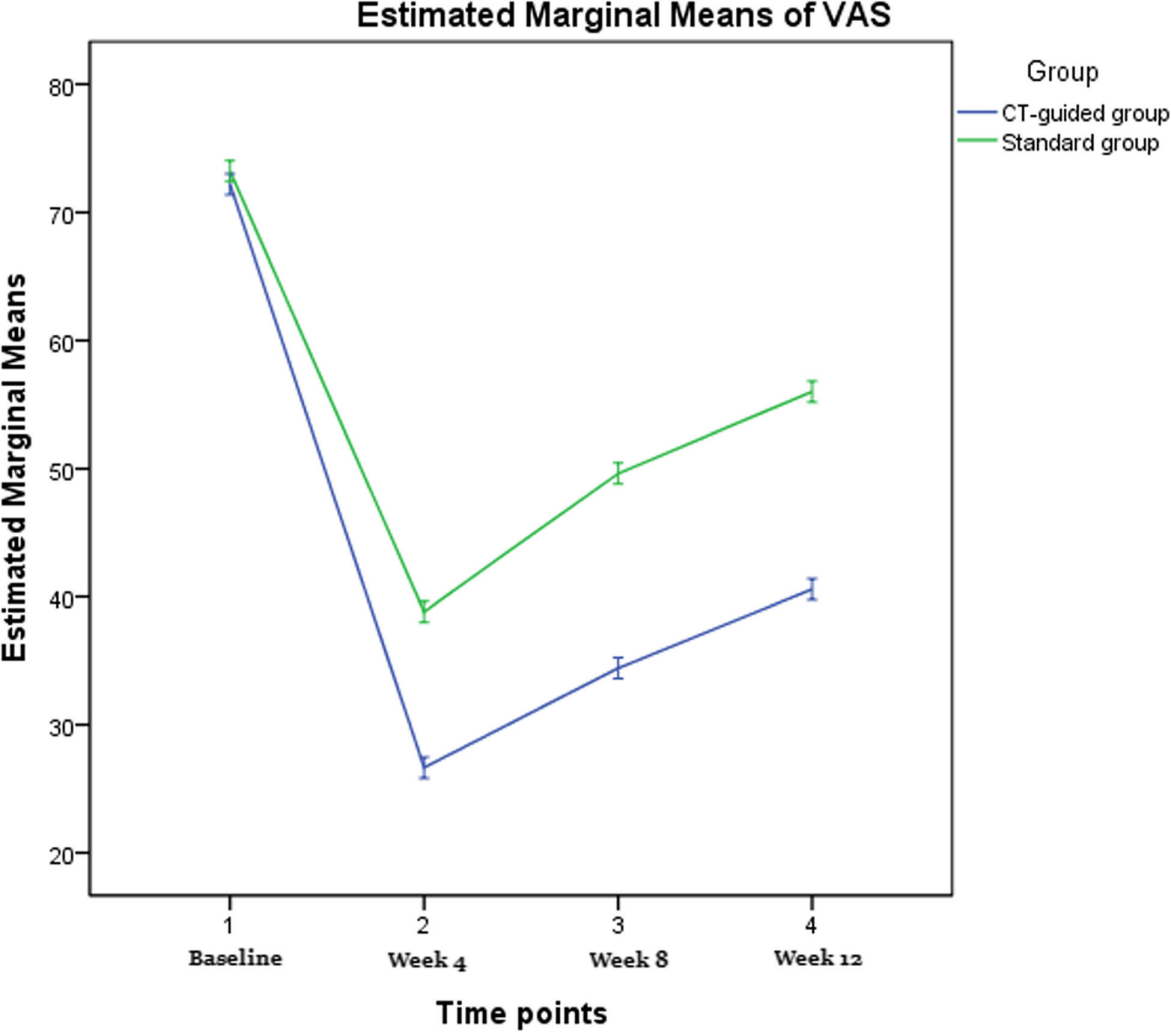
Visual analogue scale score mean profile graph using linear mixed model

The VAS scores decreased significantly in the two groups at all of the follow-up points compared to baseline values (*p* < 0.001 for all comparisons). There was no significant difference in VAS score between both groups at baseline (*p* = 0.380). The VAS scores were lower in the CT-guided group compared to the standard group at 1, 4, and 12 weeks after the procedure (Fig. 5). However, there was a difference between the two groups at week 4, week 8 and week 12 (Fig. 5). Using a linear mixed model, we determined that there was a clear difference in the average change of VAS score over time between the two groups (*P* < 0.001), as shown in Fig. 5. As a result of comparing between the two groups at each time point, we found that there was a significant difference at week 4 (*P* < 0.001), week 8 (*P* < 0.001), and week 12 (*P* = 0.001).

**Fig. 6:**
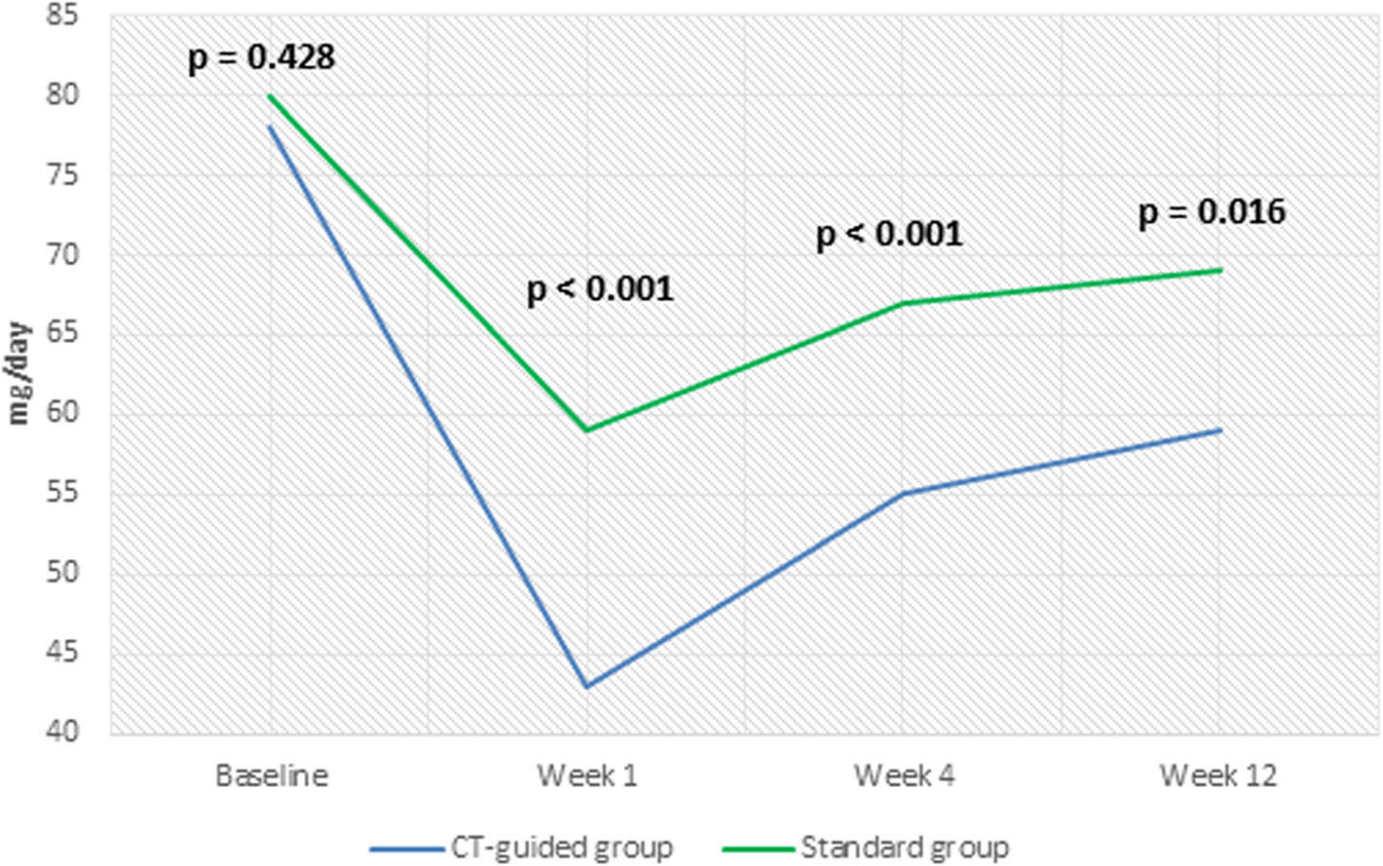
Oxycodone Consumption

**Fig. 7:**
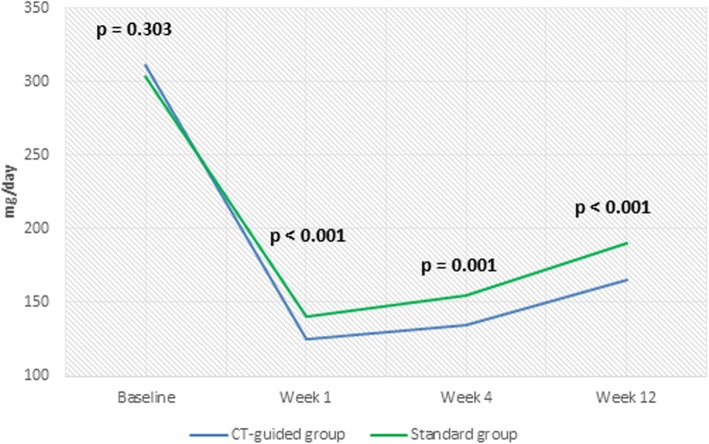
Pregabalin Consumption

Oxycodone and pregabalin consumption decreased in the two groups at all of the follow-up points compared with the baseline values. There was a significant difference between the two groups in oxycodone and pregabalin consumption (Figs. 6 and 7).

**Fig. 8:**
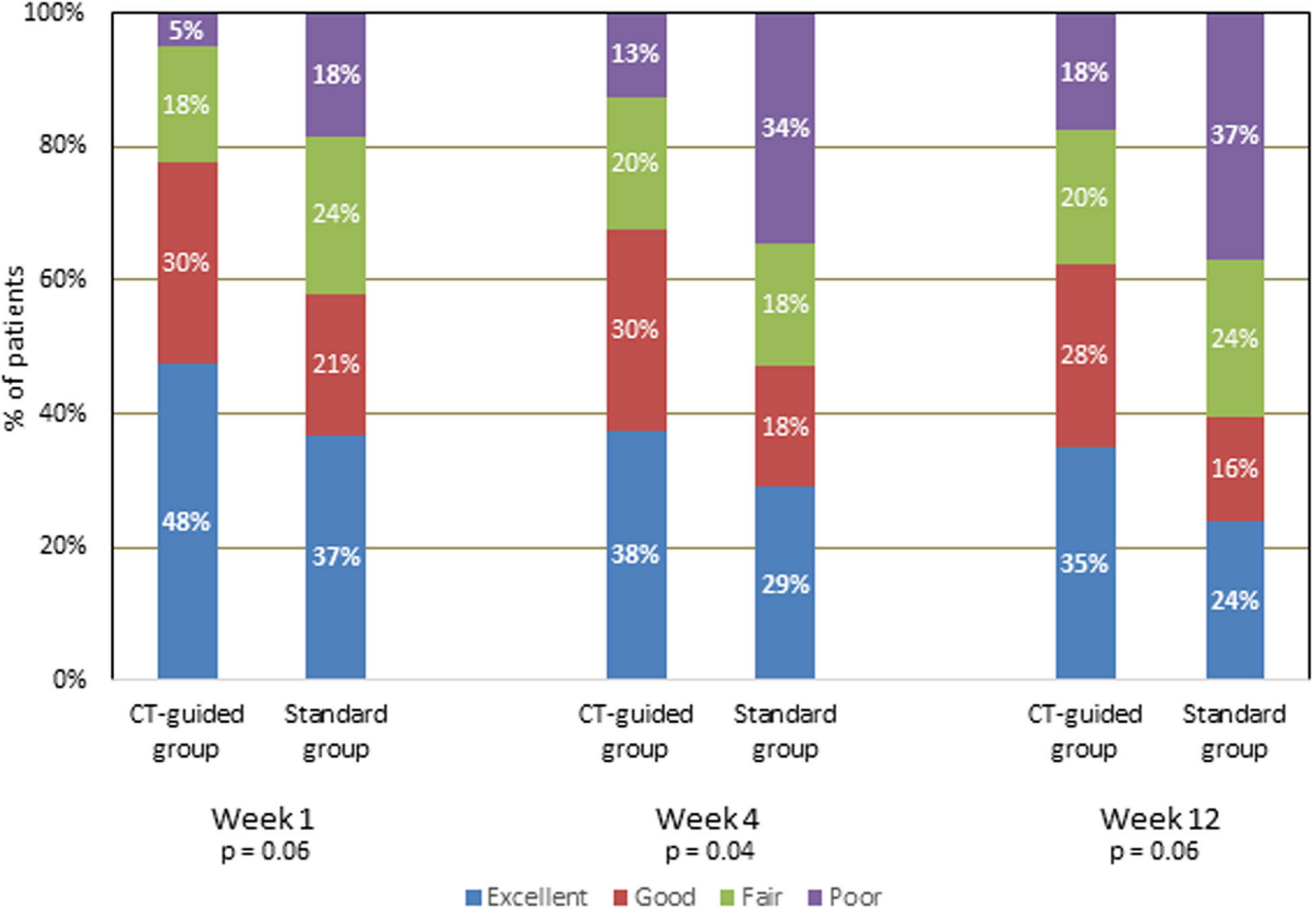
Functional improvement

Functional improvement (FI) was better (nearly significant) in the CT-guided group at weeks 1 and 4, while it was significant at week 12 (Fig. 8).

**Fig. 9:**
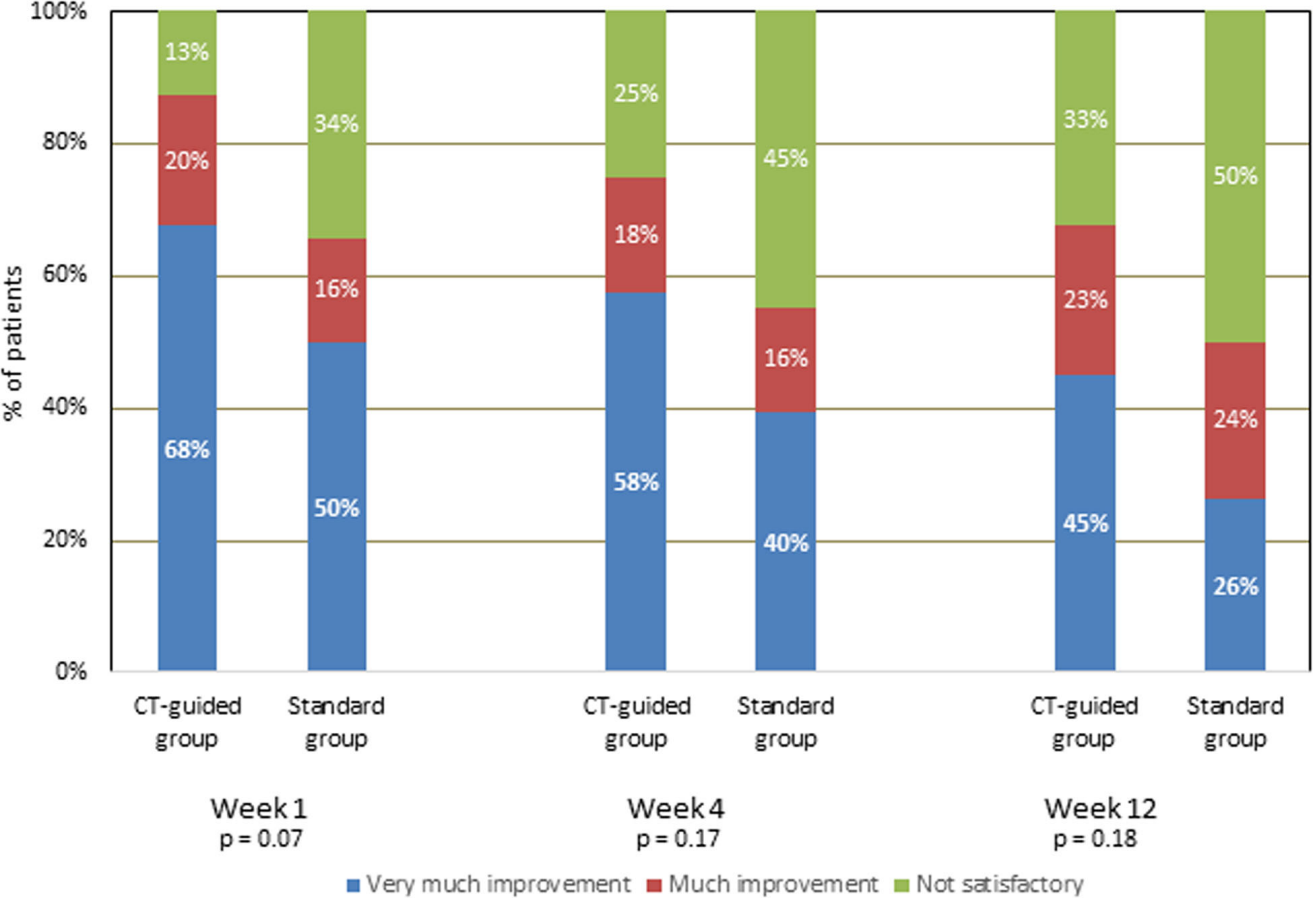
Patient global impression of changes (PGIC)

Patient satisfaction (PGIC) showed a higher indication of improvement at weeks 1, 4, and 12, but these were not statistically significant apart from week 1 which was nearly significant (Fig. 9).

The per-patient adverse events occurrence was significantly lower in the CT-guided group (*p* = 0.027) (Table 4). No infection, motor deficits, or pneumothorax were recorded.

**Table 4:** adverse effects in both groups

Adverse effects	CT-guided group *n* = 40	Standard group *n* = 38	*p* value
Minor complications			
Back pain	4 (10.0)	5 (13.2)	0.734
Soreness	7 (17.5)	8 (21.1)	0.691
Hematoma	1 (2.5)	2 (5.3)	0.610
Major complications			
Neuritis	3 (7.5)	8 (21.1)	0.086
Sensory deficits	3 (7.5)	5 (13.2)	0.476
Anesthesia dolorosa	1 (2.5)	1 (2.6)	1.000
*Per-patient adverse effects	6 (15)	14 (36.8)	0.027

Data are supplied as number and frequencies.

## Discussion

The current study demonstrated that selective TRF rhizotomy of thoracic DRG through the inferior transforaminal approach in intractable chest pain cases associated with cancer seems to be of better efficacy if performed under the combined guidance of a CT scan and fluoroscopy (Given the improvement in VAS and FI, and the higher percentages of “very much improvement” in Group C in Weeks 1, 4, and 12, that it is quite likely that the lack of statistical significance for the PGIC was due to insufficient power stemming from the small sample size).

Interventional pain procedures are indicated for refractory pain when analgesic drugs are ineffective or associated with intolerable side effects [13]. In this study, we performed TRFA at the transforaminal station adjacent to the DRG assuming its superiority to many interventions. The transforaminal approach may be considered target-specific (i.e. adjacent to DRG), which allows a lower risk of inadvertent dural puncture [14]. It also demonstrated therapeutic values in managing radicular pain in many clinical trials [15]. Lastly, the selected dermatomal segment is only addressed in cases of TRF-DRG without the need to cover the segments above and below as performed in intercostal nerve blocks (the overlap phenomenon).

Paravertebral and intercostal nerve lesioning are efficient and simple procedures that can be performed at the bedside without guidance [16]. They have many drawbacks, such as short-lived, should be repeated [17], lower analgesic efficacy [18], and pneumothorax [13, 16]. Unlike the relative constant position of DRG in the neural foramen, peripheral nerve lesioning may be misplaced in cases of tumor infiltration. Moreover, it may induce deafferentation pain and miss a proximal pain generator [19].

Dorsal chemical rhizotomy (epidural or intrathecal) may provide satisfactory analgesia for patients with lung cancer pain. However, it is associated by uncontrolled intraspinal spread and high risk for neurological deficits which ultimately limits its use in clinical practice [13, 20].

Although PCC is efficient in cancer-related chest pain [21] it has many limitations. For example; technical difficulty, 3% mortality, 11% motor weakness, mirror-image dysesthesia, and respiratory, hemodynamic, bladder and sexual dysfunction are all barriers against its widespread use [22]. Similarly, intrathecal therapy and neuromodulation are expensive requiring high standards of aftercare, which greatly limit their use in developing countries. Aside from limited life expectancy, immune compromise and neutropenia, radiation field interference and the possibility of neoplastic epidural invasion are special limitation in cancer patients [23].

Numerous anatomical barriers against the transforaminal approach in the thoracic spine have been previously described [7, 9]. For accurate RF-DRG application at the T7 level and above, van Kleef et al. created small holes in the laminae of thoracic vertebrae using 14G Kirschner wires to get into the vicinity of the thoracic intervertebral foramina [24]. A CT-scan might provide more precision in locating the thoracic DRG. This can explain the favorable results of combined CT/fluoroscopy guidance in the current study.

The authors assume that the technique of an inferior transforaminal approach may be superior to previously described approaches, e.g. the approach by Charles Gauci [25]. First, the current approach entails less bony contact and periosteal irritation, hence, less patient discomfort. Second, there is little risk of pneumothorax (no pneumothorax cases have been documented in our work).Third, the concept of inferior suprapedicular approach and Kambin’s safe triangle achieves greater validity [26] due to several proposed benefits such as avoiding injury to DRG in the superior neural foramen [27]. The peridural membrane of the suprapedicular canal has an evident nociceptive role in a manner similar to what happens in the inflamed synovium or periosteum in the case of joint or bone pain [28].

In the current study, T2-T8 levels were selected to avoid the catastrophic vascular events that may occur below the T8 due to vascular insult of the artery of Adamkiewicz, “which is the main blood supply of the anterior spinal cord below T8 [29]. Murthy et al. have identified the position of the artery of Adamkiewicz using digital subtraction angiography in the upper half of the neural foramen in 97% of patients and absent in the lower 10% of the foramen [30]. Moreover, the T2 to T8 levels - in general - are the segmental dermatomes commonly affected by chest pain pathologies.

The neuroablative RF was applied to the DRG and not the pulsed (neuromodulatory) RF for many reasons. Pulsed RF is for short-term pain relief [31], and its neuromodulatory mechanisms take 3–4 weeks to work, which is too long for cancer patients with intractable pain [32]. In addition, TRF has been postulated to be more efficient for different types of pain, e.g., idiopathic trigeminal neuralgia [33], glossopharyngeal therapy for oropharyngeal cancer [34], and facetal medial branch block [11]. Nonparticulate steroids (betamethasone) were used in conjunction with lidocaine before TRF to reduce the occurrence of neuritis (its incidence was 7.5% in the C group versus 32.5% in van Kleef’s work). Steroids have potential analgesic effects [35] while lidocaine was used for its analgesic, vasodilator and neuroprotective properties.

In the current work, the extra-guidance technique using the Xper CT reflected a better efficacy with a moderate increase of irradiation hazards (2 folds increase in ED). The implication of CT as a guidance tool has been used for interventional pain procedures, such as celiac plexus and lumbar sympathetic blocks [36, 37]. It allows better visualization of the whole needle path, surrounding soft tissues, and vascular and bony structures [38]. However, CT-associated exposure to excessive irradiation may induce carcinogenesis, genetic mutation and several other hazards [37, 38]. In previous studies conducted by Hoang et al. [39] and Schmid et al. [40] comparing irradiation dose during pain interventions guided by fluoroscopy versus CT, Hoang et al. reported 4 times more radiation exposure during CT than fluoroscopy [39]. In recent work published by Maino et al. [37] in 2018, the ED for lumbar facetal and transforaminal injections was calculated to be 8 to 10 times higher under CT versus fluoroscopy. The lower ED in the current study (2 folds) could be attributed to Xper CT guide technique for fast and accurate RF needle positioning. CT guidance is absolutely contraindicated in pregnant and pediatrics populations; however it improves anatomic localization, technical precision, post-procedures outcome and lessens the complications rate, hence it is widely practiced in many interventional procedures such as CT guided PCC, thoracic DRG-RF and many other techniques according to the clinical situation and physician judgment.

**In conclusion**, the integrated guidance of Xper CT-scan and fluoroscopy may improve the efficacy of TRF of thoracic DRG for the treatment of intractable chest pain in cancer patients. According to the authors’ knowledge, this is the first RCT studying the efficacy and safety of this technique for thoracic DRG-TRF in intractable chest cancer pain. Moreover, we stressed on the inferior transforaminal suprapedicular technique in the thoracic spine region.

**Limitations and recommendations,** this study is a single center work and single blinded hence multi-centric meta-analysis is recommended with a larger sample size of patients for verification of the data of the current work. Furthermore, a longer duration of follow up might be helpful for better evaluation and stratification of guidelines in treating lung cancer pain. Surgical confidence was not considered in the current work and should be respected in further upcoming researches.

## Data Availability

The datasets used and/or analysed during the current study are available from the corresponding author on reasonable request.

## Abbreviations

ANOVA: Analysis of variants
ASA: American society of Anesthesiologists
CT: Computerized Tomography
DRG: Dorsal root ganglia
ED: Estimated dose of irradiation exposure
EKG: Electrocardiogram
FI: Functional improvement
IV: Intravenous
mGy: Milligray
NIBP: Non-invasive blood pressure
PA: Postero-anterior
PCC: Pecutaneous cervical cordotomy
PGIC: Patient global impression of changes
PRF: Pulsed radiofrequency
RF: Radiofrequency
TDRG: Thoracic dorsal root ganglia
TRFA: Thermal radiofrequency ablation
VAS: Visual analog scale
